# Supplementary material for: Prefoldin function links meiotic chromosome segregation with cellular remodeling and reveals tubulin sensitivity of the meiotic spindle
Source: bioRxiv. 2026 May 20:2026.05.20.726416. Preprint. [Version 1] doi: 10.64898/2026.05.20.726416 (PMC13228207; doi:10.64898/2026.05.20.726416)

bioRxiv preprint doi: https://doi.org/10.64898/2026.05.20.726416; this version posted May 20, 2026. The copyright holder for this preprint (which was not certified by peer review) is the author/funder, who has granted bioRxiv a license to display the preprint in perpetuity. It is made available under a CC-BY 4.0 International license.

Supplementary Figure Legends

Supplementary Figure 1. CRISPR screening and following experiments identify Gim3 as meiotic regulator

(A) Screening scheme. A CRISPEY library targeting short open reading frames (sORFs) and truncated ORFs (tORFs) was constructed and introduced into yeast. After meiosis induction, spores were enriched by zymolyase treatment, followed by plasmid extraction, library preparation, and next-generation sequencing.

(B) Enrichment scores for resistance to zymolyase treatment. Random sequences are shown in blue and positive control sequences are shown in red.

(C) Validation of top sORF hits identified in (B). Deletion strains for the indicated sORFs were constructed, and meiotic progression was assessed by DAPI staining at 6 hours in SPO. The y-axis represents the normalized proportion of mono-nucleated cells. N = 2-3; Dunnett's test.

bioRxiv preprint doi: https://doi.org/10.64898/2026.05.20.726416; this version posted May 20, 2026. The copyright holder for this preprint (which was not certified by peer review) is the author/funder, who has granted bioRxiv a license to display the preprint in perpetuity. It is made available under a CC-BY 4.0 International license.

More than 100 cells were quantified per replicate.

(D) Genomic locus of *GIM3* and *sORF2*. *sORF2* resides within the *GIM3* locus and is transcribed in the reverse direction.

(E) Complementation assay. *gim3*Δ strains were transformed with plasmids expressing either *GIM3* or *sORF2*, and meiotic progression was assessed by DAPI staining. The y-axis represents the normalized proportion of mononucleated cells at 6 hours in SPO. N = 3. Dunnett's test. More than 100 cells were quantified per replicate.

(F) Spore colony growth of WT, *gim3*Δ with empty cassette, and *gim3*Δ with *GIM3* or *sORF2* complementation after 2 days at 30 °C (left). Quantification of spore viability (right). N = 3; data are represented as mean ± SD. Dunnett's test. 64-80 spores were quantified per replicate. The results for WT, *gim3*Δ carrying the empty cassette, and *gim3*Δ complemented with *GIM3* are identical to those shown in Figure 2C, D.

Supplementary Figure 2. Chromatin mis-segregation was not detected during mitosis in *gim3*Δ cells grown in pre-sporulation or synthetic media

(A) Quantification of chromatin mis-segregation during mitosis in WT and *gim3*Δ cells in BYTA. Htb1-mCherry imaging was performed every 10 min during mitosis. N = 3; ND, not detected; 71-80 cells were quantified per replicate.

(B) Quantification of chromatin mis-segregation during mitosis in WT and *gim3*Δ cells in synthetic media. Htb1-mCherry imaging was performed every 15 min during mitosis. N = 3; ND, not detected; 27-55 cells were quantified per replicate.

(C) Live-cell imaging of Htb1-mCherry in *tpk1-as tpk2*Δ *tpk3*Δ cells (top) and *gim3*Δ *tpk1-as tpk2*Δ *tpk3*Δ cells (bottom) in YPD treated with 1NM-PP1 and rapamycin. Cells were imaged every 15 min during meiosis. Yellow arrowheads indicate chromatin mis-segregation. Time is shown relative to the onset of anaphase I (0 min). Scale bars, 3 μm

(D) Quantification of Htb1-mCherry mis-segregation events during MI (left) and MI&MII (right). N = 4; data are represented as mean ± SD; t-test. 100-130 cells were quantified per replicate.

Supplementary Figure 3. Identification of Gim3 interactors and functional assessment of prefoldin subunits

(A) Mass spectrometry analysis of immunopurified proteins using Gim3 as bait in mitotic cells. The plot shows enrichment scores (x-axis, Gim3 IP versus control IP) versus $-\log_{10}$ P-value (y-axis). Gim3 itself is highlighted in red, and other prefoldin subunits are highlighted in green.

(B) Venn diagram showing the overlap of proteins identified in mitotic and meiotic (5 hours in SPO).

(C) List of proteins identified in the co-purification experiments under mitotic and meiotic conditions. Proteins are grouped as common, mitosis-specific, or meiosis-specific interactors,

bioRxiv preprint doi: https://doi.org/10.64898/2026.05.20.726416; this version posted May 20, 2026. The copyright holder for this preprint (which was not certified by peer review) is the author/funder, who has granted bioRxiv a license to display the preprint in perpetuity. It is made available under a CC-BY 4.0 International license.

corresponding to the Venn diagram in panel B.

(D) Growth curves as assessed by OD_{600} measurement of mitotic cells carrying deletions in prefoldin subunits (*gim1*Δ, *gim2*Δ, *gim4*Δ, *gim5*Δ, and *gim6*Δ) versus WT controls. Growth was monitored in YPD; N = 2-3; data are represented as mean ± SD.

Supplementary Figure 4. Tub1 protein levels and spindle dynamics in prefoldin subunit deletion mutants

(A) Ribosome profiling analysis during meiosis (4 hours in SPO). Positional ribosome footprint profiles are shown for *TUB1*, and normalized read counts are shown for *TUB1*, *TUB2*, *TUB3*, and *ACT1* in WT and gim3Δ cells.

(B) Western blot analysis of Tub1 protein levels in mitotic cells of WT and individual prefoldin subunit deletion strains (*gim1*Δ, *gim2*Δ, *gim4*Δ, *gim5*Δ, and *gim6*Δ). Hxk2 was used as a loading control. N = 3; data are represented as mean ± SD; Dunnett's test.

(C) Tub1 protein levels in WT and individual prefoldin subunit deletion strains (*gim1*Δ, *gim2*Δ, *gim4*Δ, *gim5*Δ, and *gim6*Δ) strains after 4 hours in SPO (meiosis). Hxk2 was used as a loading control. N = 3; data are represented as mean ± SD; Dunnett's test.

(D) Representative western blot of Tub1 in WT and *gim3*Δ cells during meiosis (left). Hxk2 (Hexokinase isoenzyme 2) was used as a loading control and Ndt80 was used as a marker of meiotic progression. Quantification of Tub1 protein levels (right). N = 3; data are represented as mean ± SD.

(E) Quantification of GFP-Tub1 intensity in WT and *gim3*Δ cells during MI and MII. Each dot represents a single cell. Bars indicate mean ± SD. Mann-Whitney U test; 32-35 cells.

Supplementary Figure 5. Rec8 cleavage dynamics and checkpoint dependence of mis-segregation in *gim3*Δ cells

(A) Quantification of chromatin mis-segregation in WT, *gim3*Δ, *mad2*Δ, and *gim3*Δ *mad2*Δ cells. N = 3; data are represented as mean ± SD; Welch's t-test.

(B) Representative western blot of Rec8 in WT and *gim3*Δ cells undergoing meiosis. Deletion of *UBR1*, which stabilizes the cleaved Rec8 fragment, was used to facilitate detection of the Rec8 cleavage product. Hxk2 was used as a loading control.

(C) Quantification of uncleaved (left) and cleaved (right) Rec8 protein levels from (B). N = 3; data are represented as mean ± SD.

Supplementary Figure 6. Reduced Tub1 levels or inhibition of tubulin polymerization leads to chromosome mis-segregation

(A) Representative western blot of Tub1 protein levels in WT, *gim3*Δ, and *TUB1/tub1*Δ strains during mitotic growth (left) and meiosis (4 hours in SPO) (right). Hxk2 was used as a loading control.

bioRxiv preprint doi: https://doi.org/10.64898/2026.05.20.726416; this version posted May 20, 2026. The copyright holder for this preprint (which was not certified by peer review) is the author/funder, who has granted bioRxiv a license to display the preprint in perpetuity. It is made available under a CC-BY 4.0 International license.

(B) Quantification of Tub1 protein levels from (A). Data are represented as mean ± SD; N = 3; Tukey's multiple comparison test.

(C) Quantification of chromatin mis-segregation during mitosis in WT and *TUB1/tub1*Δ cells in YPD. N = 3; ND, not detected; Welch's t-test; 64-86 cells were quantified per replicate.

(D) Quantification of chromatin mis-segregation during mitosis in WT and *TUB1/tub1*Δ cells in BYTA. N = 3; data are presented as mean ± SD; Welch's t-test; 66-104 cells were quantified per replicate.

(E) Quantification of chromatin mis-segregation during mitosis in WT and *TUB1/tub1*Δ cells in synthetic media. N = 3; data are presented as mean ± SD; Welch's t-test; 24-44 cells were quantified per replicate.

(F) Spore colony growth of tetrads derived from WT and *Tub1/tub1*Δ strains on YPD after 2 days at 30 °C (left). Quantification of spore viability (right). N = 3; data are represented as mean ± SD; Welch's t-test. 80 spores were quantified per replicate.

(G) Representative western blot of Tub1 protein levels in WT, *gim3*Δ and *gim3*Δ overexpressing *TUB1* strains during meiosis (4 hours in SPO). Hxk2 was used as a loading control.

(H) Quantification of Tub1 protein levels from (G). Data are represented as mean ± SD; N = 3.

(I) Normalized Act1 protein levels in WT and *gim3*Δ cells at 3, 4, 5 and 6 hours in SPO, quantified by mass spectrometry. Mass spectrometry analysis was performed once for each time point.

(J) Quantification of chromatin mis-segregation events in WT cells, *gim3*Δ cells carrying the empty cassette, and *gim3*Δ cells expressing *TUB1* or *ACT1*, based on live-cell imaging of Htb1-mCherry. *TUB1* or *ACT1* expression was driven by a GAL promoter and induced using the Gal4-ER system upon addition of β-estradiol (Gao and Pinkham, 2000). β-estradiol was added at 2.5 hour in SPO to a final concentration of 1 nM. N = 3; data are represented as mean ± SD; Tukey's multiple comparison test. 112-158 cells were quantified per replicate.

(K) Quantification of spore viability in WT cells, *gim3*Δ cells carrying the empty cassette, and *gim3*Δ cells expressing *TUB1* or *ACT1*. *TUB1* or *ACT1* expression was induced from a GAL promoter using the Gal4-ER system by addition of 1 nM β-estradiol at 2.5 hour in SPO. Spore viability was determined by tetrad dissection. N = 3; data are represented as mean ± SD; Tukey's multiple comparison test.

(L) Normalized Tub2 protein levels in WT and *gim3*Δ cells at 3, 4, 5 and 6 hours in SPO, quantified by mass spectrometry. Mass spectrometry analysis was performed once for each time point.

(M) Quantification of chromatin mis-segregation events in WT, *gim3*Δ, and *gim3*Δ *TUB2/tub2*Δ cells, based on live-cell imaging of Htb1-mCherry. Chromatin mis-segregation was scored using Htb1-mCherry. N = 3; data are represented as mean ± SD; Tukey's multiple comparison test. 101-189 cells were quantified per replicate.

bioRxiv preprint doi: https://doi.org/10.64898/2026.05.20.726416; this version posted May 20, 2026. The copyright holder for this preprint (which was not certified by peer review) is the author/funder, who has granted bioRxiv a license to display the preprint in perpetuity. It is made available under a CC-BY 4.0 International license.

Supplementary Figure 7. Nuclear component inheritance and organelle remodeling in *gim3*Δ cells during meiosis

(A) Quantification of Nup170-GFP inheritance at 21 hours in SPO. N = 3; data are represented as mean ± SD; t-test. 83-140 cells were quantified per replicate.

(B) Live-cell imaging of WT and *gim3*Δ cells expressing Htb1-mCherry and Nup170-GFP during meiosis. Yellow arrowheads indicate degradation of chromatin mass with Nup170-GFP. Time is shown relative to the onset of anaphase II (0 min). Scale bars, 3 µm.

(C) Quantification of degradation of chromatin mass with Nup170-GFP shown in (B). N = 3; data are represented as mean ± SD; Welch's t-test. 63-115 cells were quantified per replicate.

(D) Live-cell imaging of WT cells expressing Htb1-mCherry and Nup170-GFP during meiosis treated with benomyl (20 µg/mL). Time is shown relative to the onset of anaphase II (0 min). Scale bars, 3 µm.

(E) Quantification of Nup170-GFP sequestration from chromatin in WT cells, gim3Δ cells carrying the empty cassette, and *gim3*Δ cells expressing *TUB1* or *ACT1*. *TUB1* or *ACT1* expression was induced from a GAL promoter using the Gal4-ER system by addition of 1 nM β-estradiol at 2.5 hour in SPO. The WT, *gim3*Δ empty-cassette, and *gim3*Δ cells expressing *TUB1* data are the same as those presented in Figure 5B and are included here for comparison. N = 3; data are represented as mean ± SD; Tukey's multiple comparison test. 56-88 cells were quantified per replicate.

(F) Quantification of Nup170-GFP sequestration from chromatin in *WT*, *gim3*Δ, and *gim3*Δ *TUB2/tub2*Δ heterozygous diploid cells. N = 3; data are represented as mean ± SD; Tukey's multiple comparison test. 77-117 cells were quantified per replicate.

(G) Live-cell imaging of WT and *gim3*Δ cells expressing Htb1-mCherry and Cit1-GFP (mitochondrial marker) during meiosis. Time is shown relative to the onset of anaphase II (0 min). Yellow arrowheads indicate detachment of mitochondria from the cell periphery. Scale bars, 3 µm.

(H) Live-cell imaging of WT and *gim3*Δ cells expressing Htb1-mCherry and HDEL-GFP (ER marker) during meiosis. Time is shown relative to the onset of anaphase II (0 min). Yellow arrowheads indicate the presence (WT) or absence (*gim3*Δ) of ER cables, and blue arrowheads indicate ER detachment from the cell periphery. Scale bars, 3 µm.

(I) Quantification of the percentage of Hsp104-GFP positive cells in WT and *gim3*Δ cells at 4 hours in SPO. Hsp104-GFP positive cells were scored based on the presence of visible Hsp104-GFP foci. N = 3; data are represented as mean ± SD; Welch's t-test. 120-189 cells were quantified per replicate.

(J) Representative images of Tub2-GFP in WT and *gim3*Δ cells at 0 hours in SPO (top) and quantification of the percentage of cells containing Tub2-GFP aggregates (bottom). Scale bars, 5 µm. N = 3; data are represented as mean ± SD. ND, not detected. More than 30 cells were

bioRxiv preprint doi: https://doi.org/10.64898/2026.05.20.726416; this version posted May 20, 2026. The copyright holder for this preprint (which was not certified by peer review) is the author/funder, who has granted bioRxiv a license to display the preprint in perpetuity. It is made available under a CC-BY 4.0 International license.

quantified per replicate.

(K) Quantification of spindle length in WT and *rec8*Δ cells during meiosis I (top) and meiosis II (bottom), measured using Spc42 as a spindle pole body marker. Spindle length was calculated as the distance between Spc42 signals. Data are represented as mean ± SD; Mann-Whitney U test. 44-47 cells were quantified per replicate.

(L) Quantification of chromatin mis-segregation events in WT, *rec8*Δ, and *pCLB2-3HA-SGO1* cells, based on live-cell imaging of Htb1-mCherry. Chromatin mis-segregation was scored using Htb1-mCherry as a chromatin marker. N = 3; data are represented as mean ± SD; Tukey's multiple comparison test. 45-109 cells were quantified per replicate.

Supplementary Figure 8. Tub1 protein level during meiosis

Tub1 protein abundance during mitosis and meiosis based on mass spectrometry data reanalyzed from (Cheng et al., 2018).

Supplementary Figure 9. Reanalysis of the association between prefoldin and human phenotypes

Data from (Pacheco et al., 2011) (A), (Platts et al., 2007) (B), and (Kui et al., 2019) (C) were reanalyzed to assess associations between prefoldin and human phenotypes.

Supplementary Figure 10. Replicated experiments and full images of gels

Supplementary Files

File S1. CRISPEY-based screening to identify functional sORF loci during meiosis

This file includes the library sequences (Tab 1), MAGeCK scores (Tab 2), and the positive control genes (Tab 3).

File S2. Mass spectrometry of Gim3 associated protein levels in mitosis or meiosis

Cell extracts were immunopurified from Gim3-3V5 cells, and protein abundance was quantified by TMT10-based measurement. Analysis was performed using Spectrum Mill software. Data from three biological replicates for each condition are shown.

File S3. Mass spectrometry of total protein levels meiotic cells in WT and *gim3*Δ

Cell extracts from WT and *gim3*Δ cells were subjected to mass spectrometry, and protein abundance was quantified by TMT10-based measurement.

File S4. Ribosome profiling of meiotic cells in WT and *gim3*Δ

RPKM values for WT and *gim3*Δ cells during meiosis (4 hours in SPO).

bioRxiv preprint doi: https://doi.org/10.64898/2026.05.20.726416; this version posted May 20, 2026. The copyright holder for this preprint (which was not certified by peer review) is the author/funder, who has granted bioRxiv a license to display the preprint in perpetuity. It is made available under a CC-BY 4.0 International license.

File S5. Strains, plasmids, and primers

This file includes all strains used in this study (Tab 1), as well as plasmids (Tab 2), and primers (Tab 3).

Supplementary figure 1

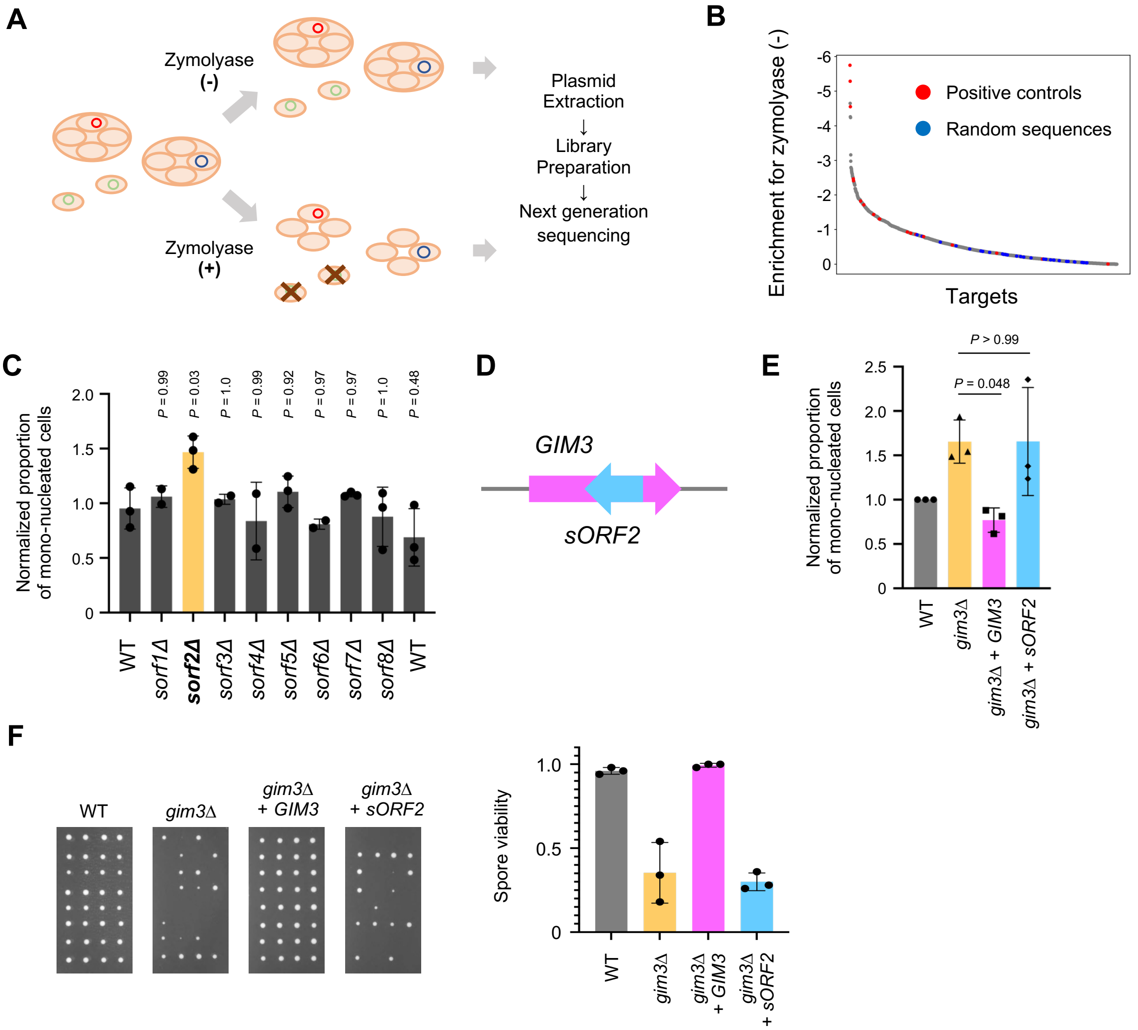

Supplementary figure 2

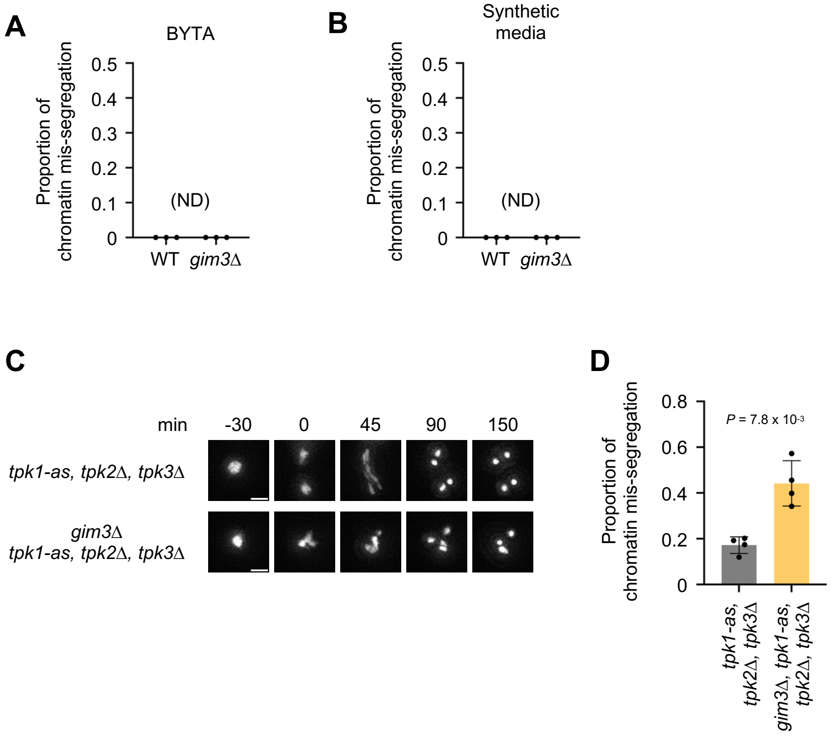

Supplementary figure 3

A

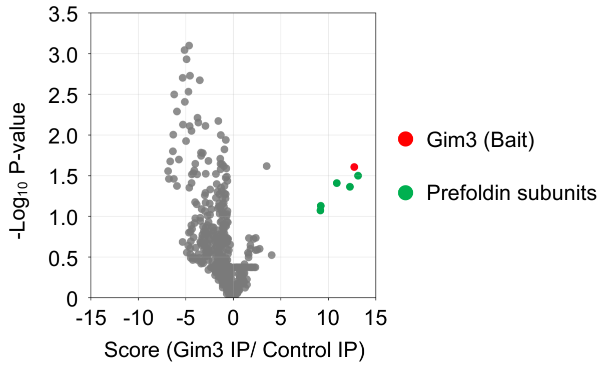

B

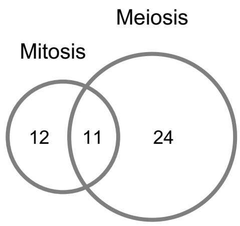

C

Common	Mitosis specific	Meiosis specific
Gim6	Vtc1	Rpl42
Gim3	Sna2	Mrps17
Gim5	Crc1	Mrps12
Gim1	Yet3	Rpl31a
Gim2	Cyb5	Hap2
Gim4	Ccct6	Pdc1
Gnd1	Tsa1	Cob
Cct4	Cct3	Rpl21b
Vma1	Cct5	Mrps9
Nce102	Ilv3	Rps9b
Act1	Sbh1	Fmc1
	Tcp1	Mrp20
		Ydr119w-a
		Sir1
		Mrpl31
		Om14
		Lcb5
		Sue1
		Cof1
		Rpl17a
		Mrp2
		Cys3
		Cit1
		Rps9a

D

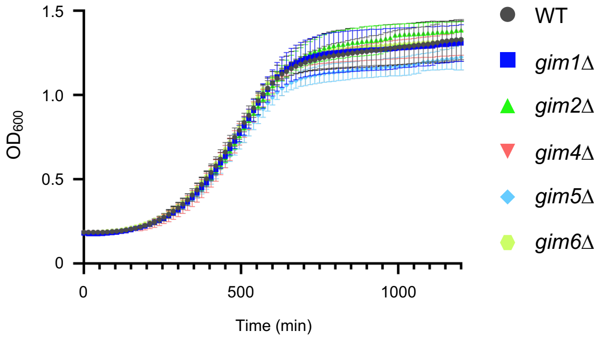

Supplementary figure 4

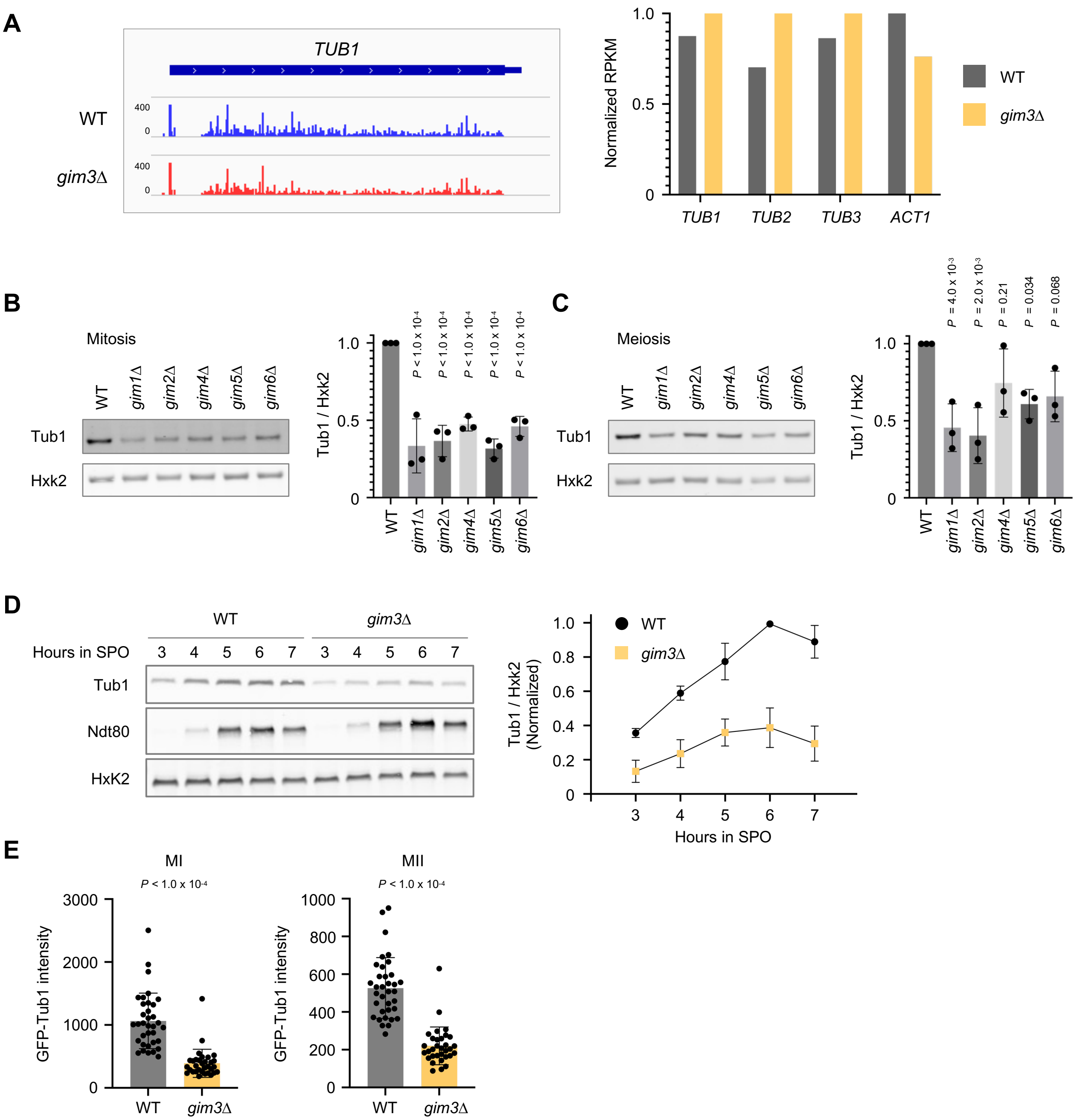

Supplementary figure 5

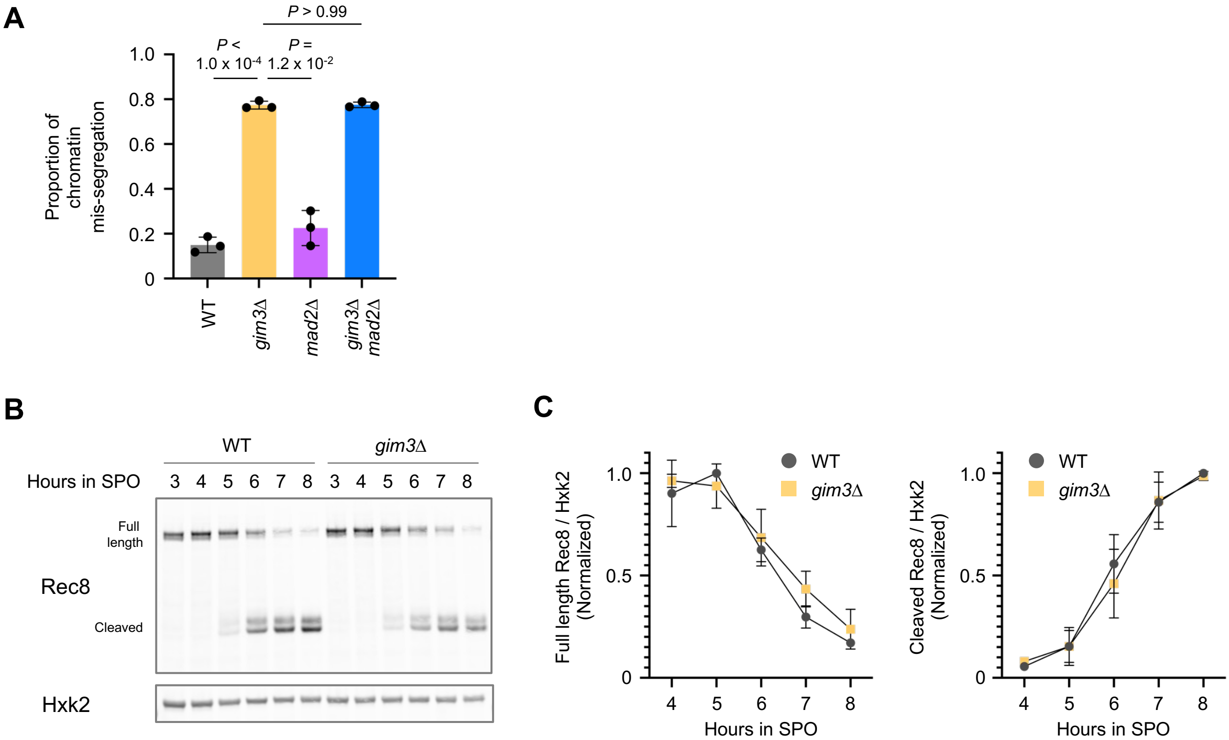

Supplementary figure 6

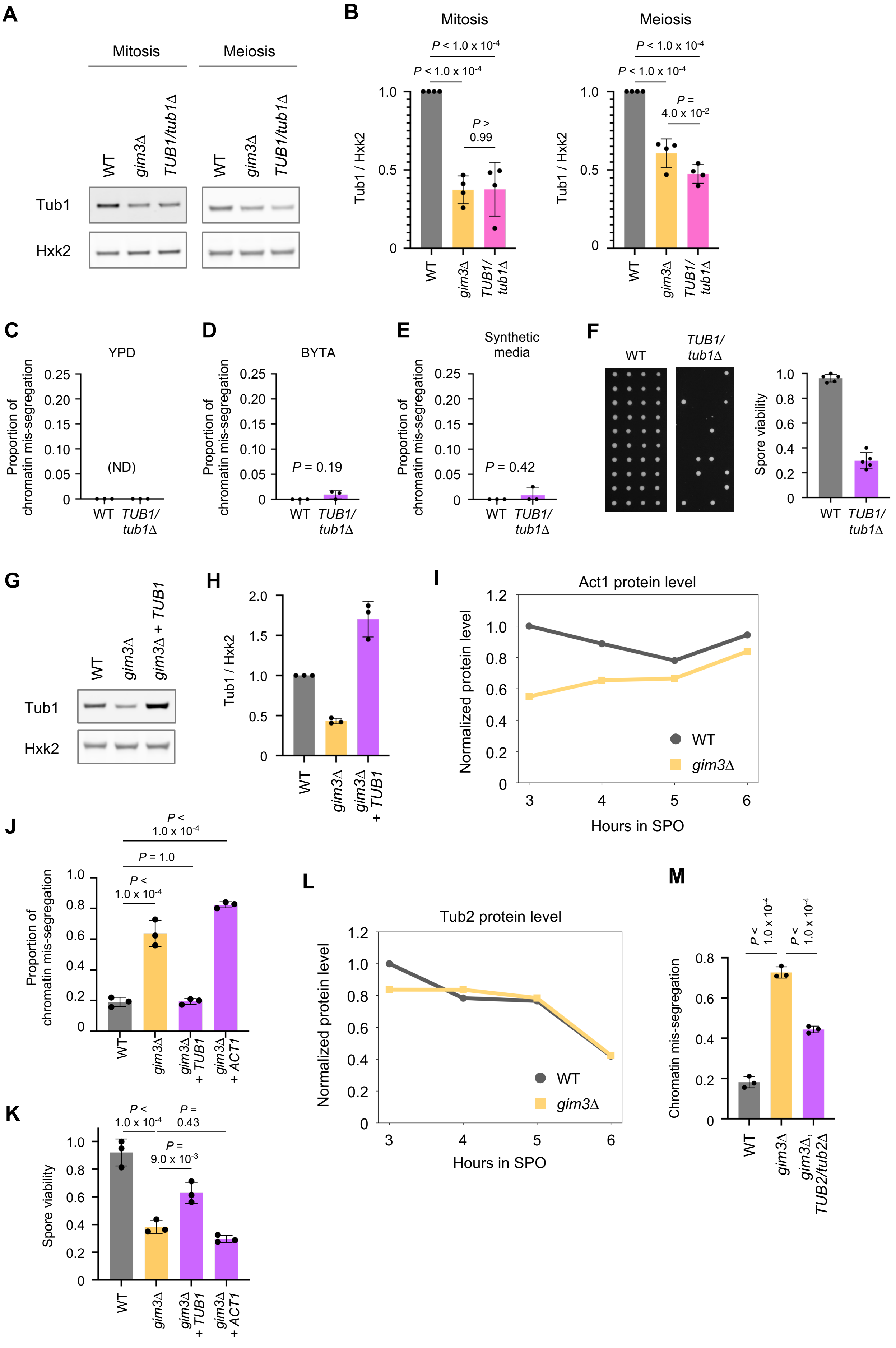

Supplementary figure 7

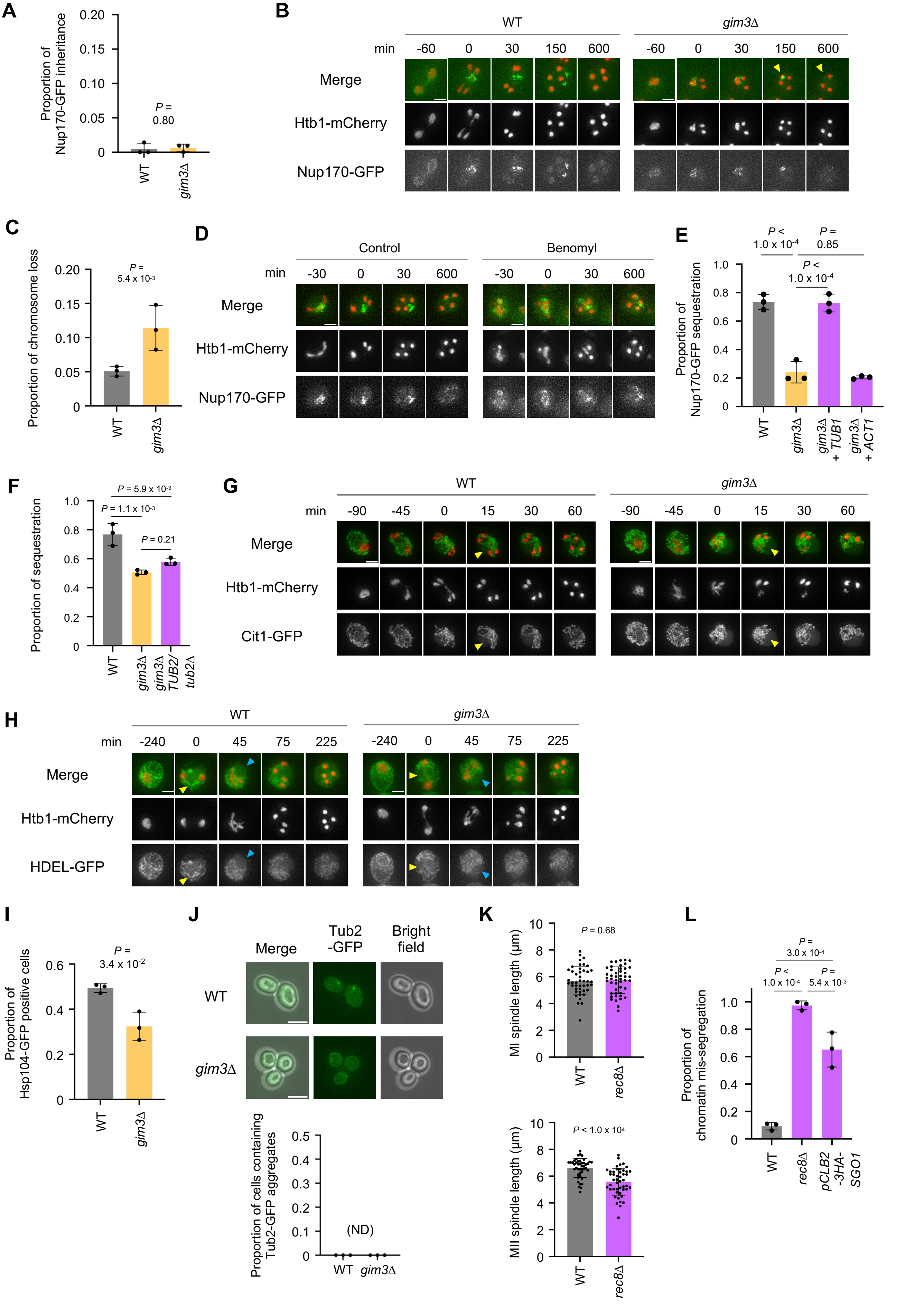

Supplementary figure 8

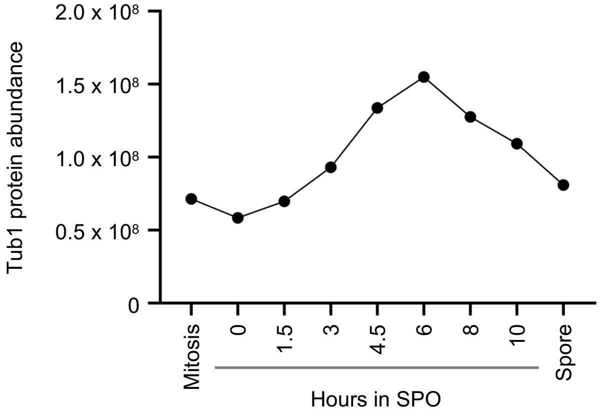

Supplementary figure 9

A Low motility sperm (from Pacheco et al., 2011)

Illumina CpG ID	Gene	NCBI	Slope (log_2)	Q-value
cg07697078	*PFDN1*	5201	0.15	0.03633
cg13576739	*PFDN6*	10471	0.14	0.03633
cg13347296	*PFDN4*	5203	0.12	0.03665
cg10107186	*PFDN5*	5204	0.17	0.03771
cg11368791	*PFDN6*	10471	0.1	0.03827

B Teratozoospermia (from Platts et al., 2007)

Probe Set ID	Gene Symbol	Gene	Ns Abundance	Tz Abundance	Fold Change Tz/Ns	P value
201507_at	PFDN1	prefoldin 1	67.62	8.68	-7.790323	0.005055
205361_s_at	PFDN4	prefoldin 4	282.49	32.35	-8.732303	0.001356
207132_x_at	PFDN5	prefoldin 5	1254.85	128.46	-9.76841	0.00008
210908_s_at	PFDN5	prefoldin 5	806.78	50.53	-15.96636	0.000051

C Spermatogenic arrest (from Kui et al., 2019)

Premeiotic arrest
down-regulated

Gene symbol	p-value
PFDN2	0.015764163
CCT4	0.069443714

Supplementary figure 10

Figure 2A

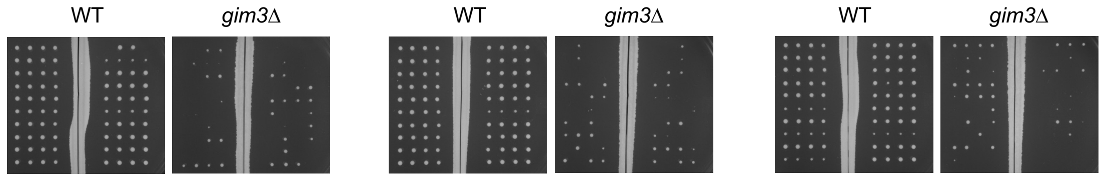

Figure 2C, S1F

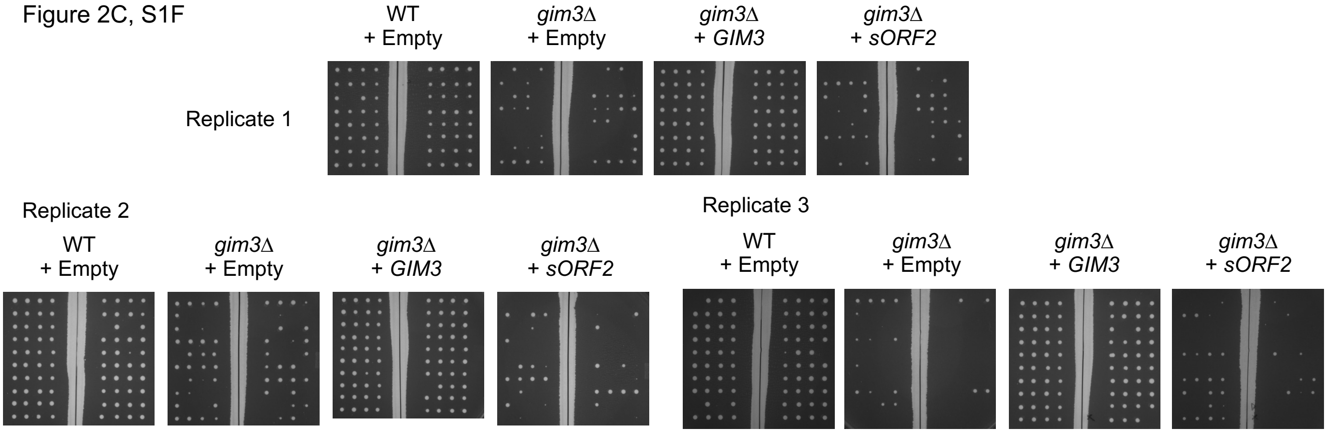

Figure 2E

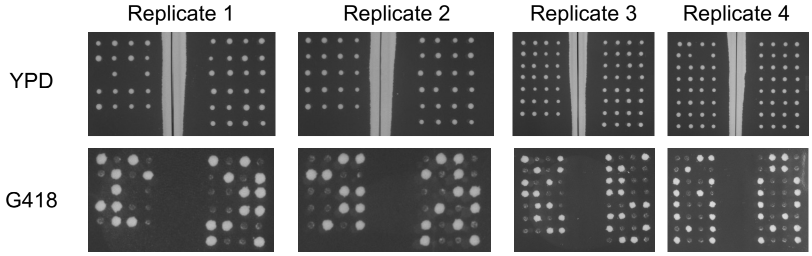

Figure 2H

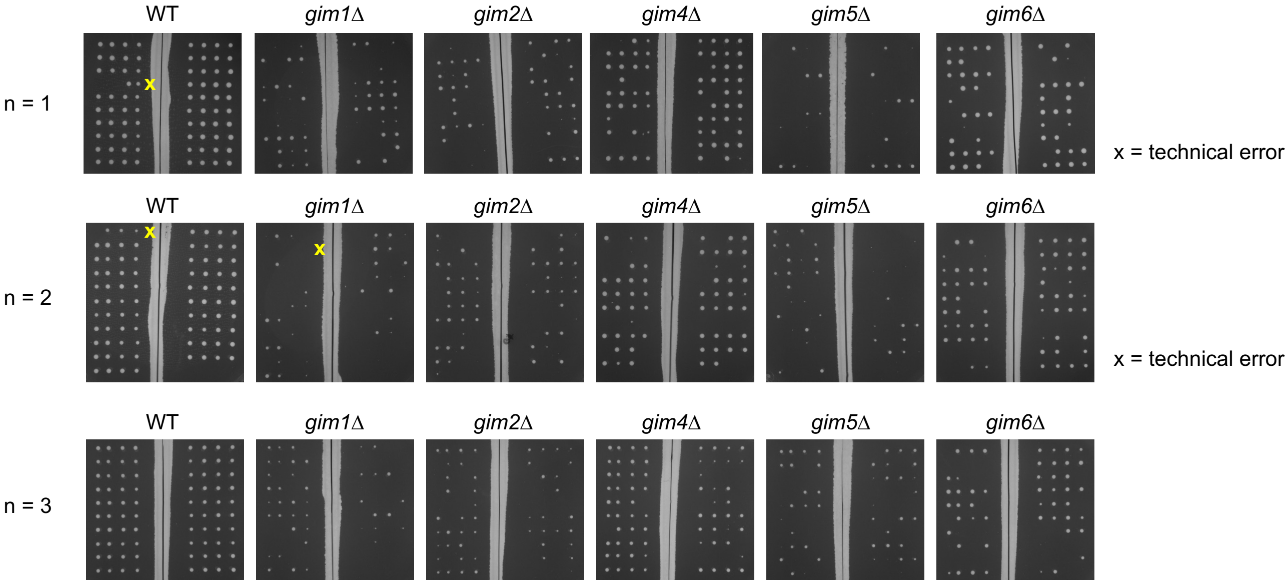

Figure 4G

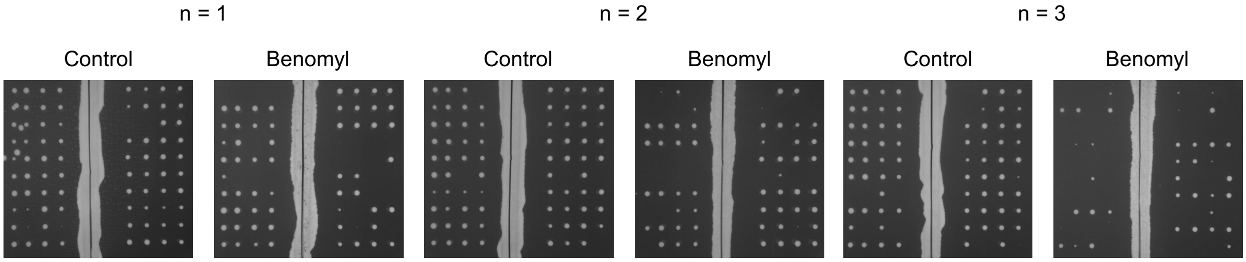

Figure S6F

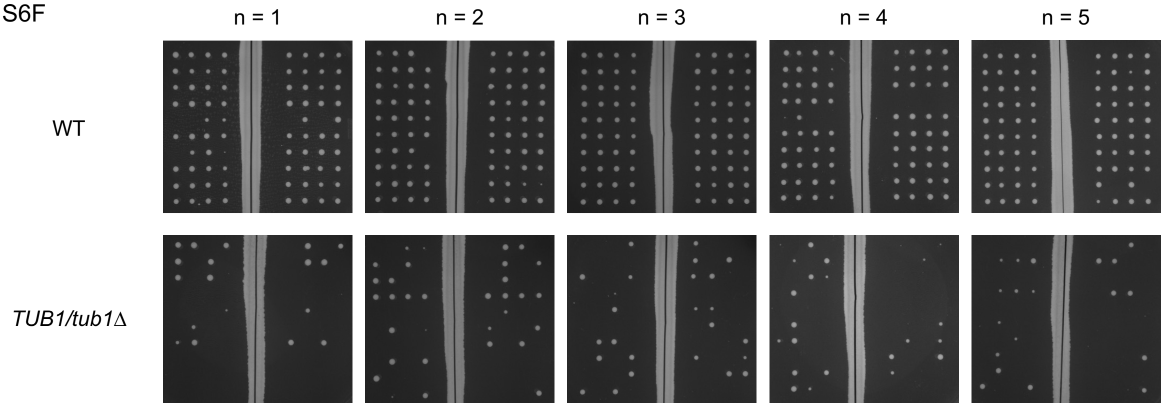

Figure 4K

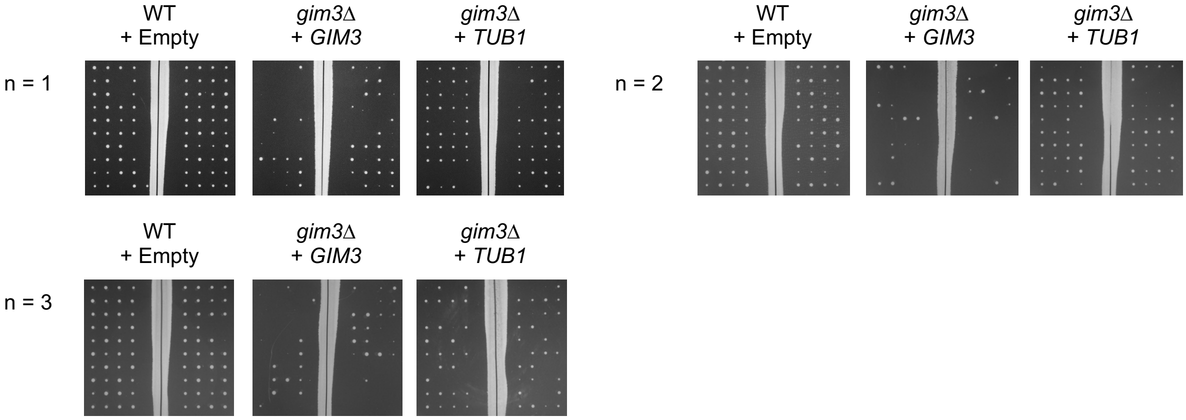

Figure S6K

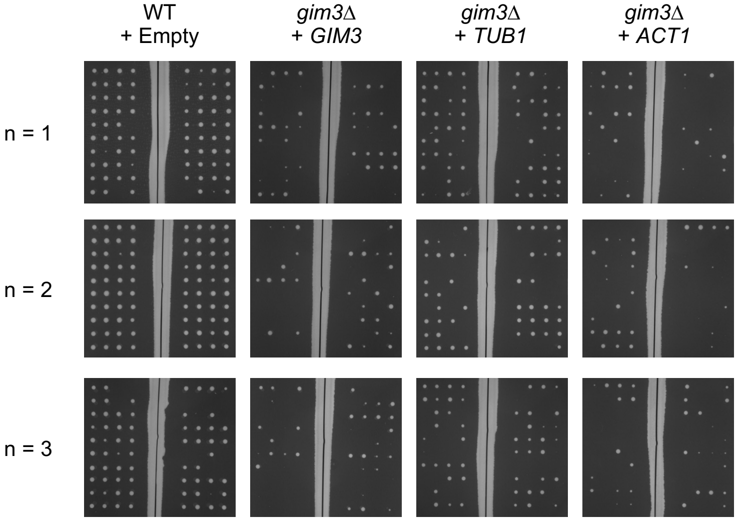

Full gel images

Figure 3D, S6A

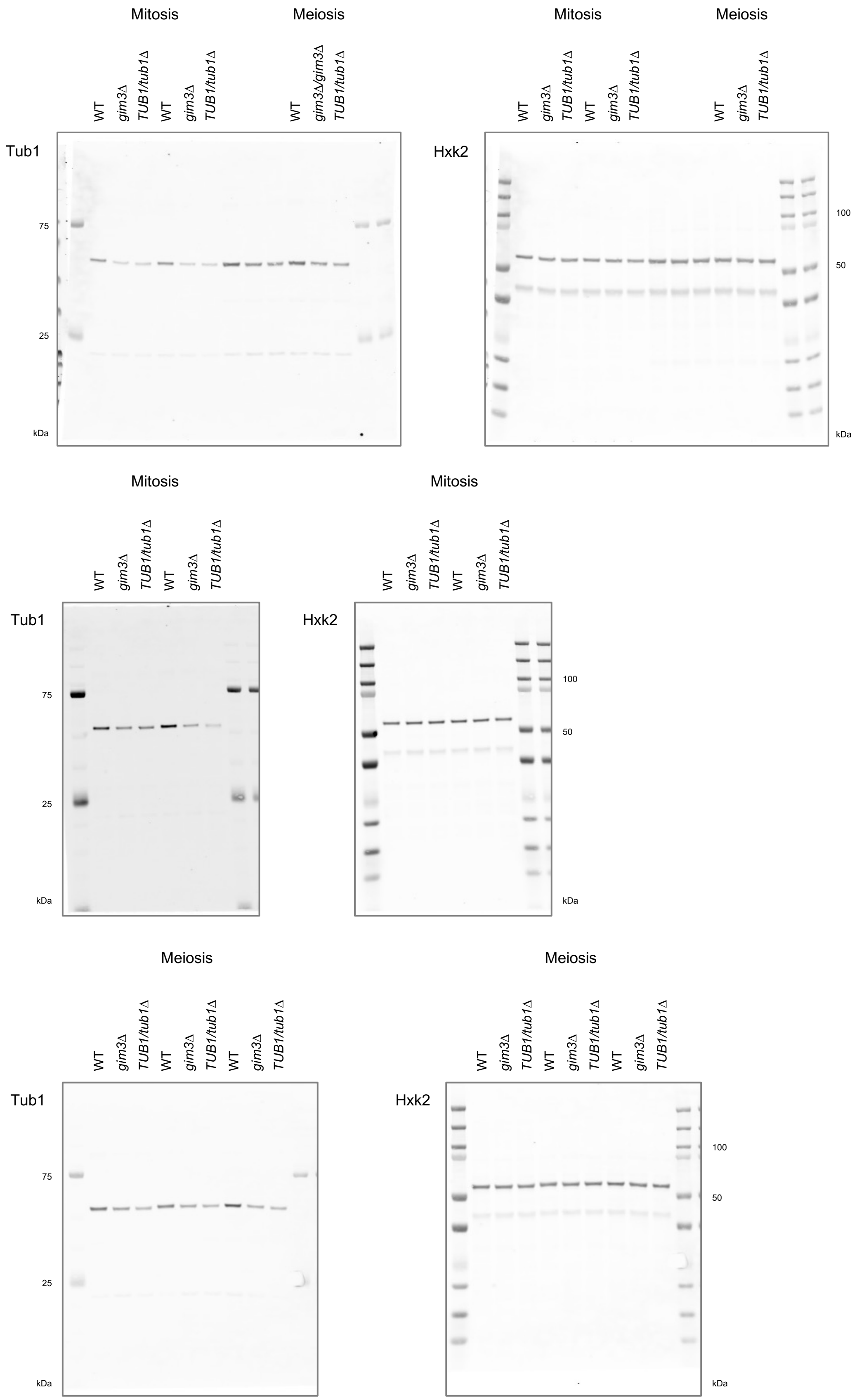

Figure S4B, C

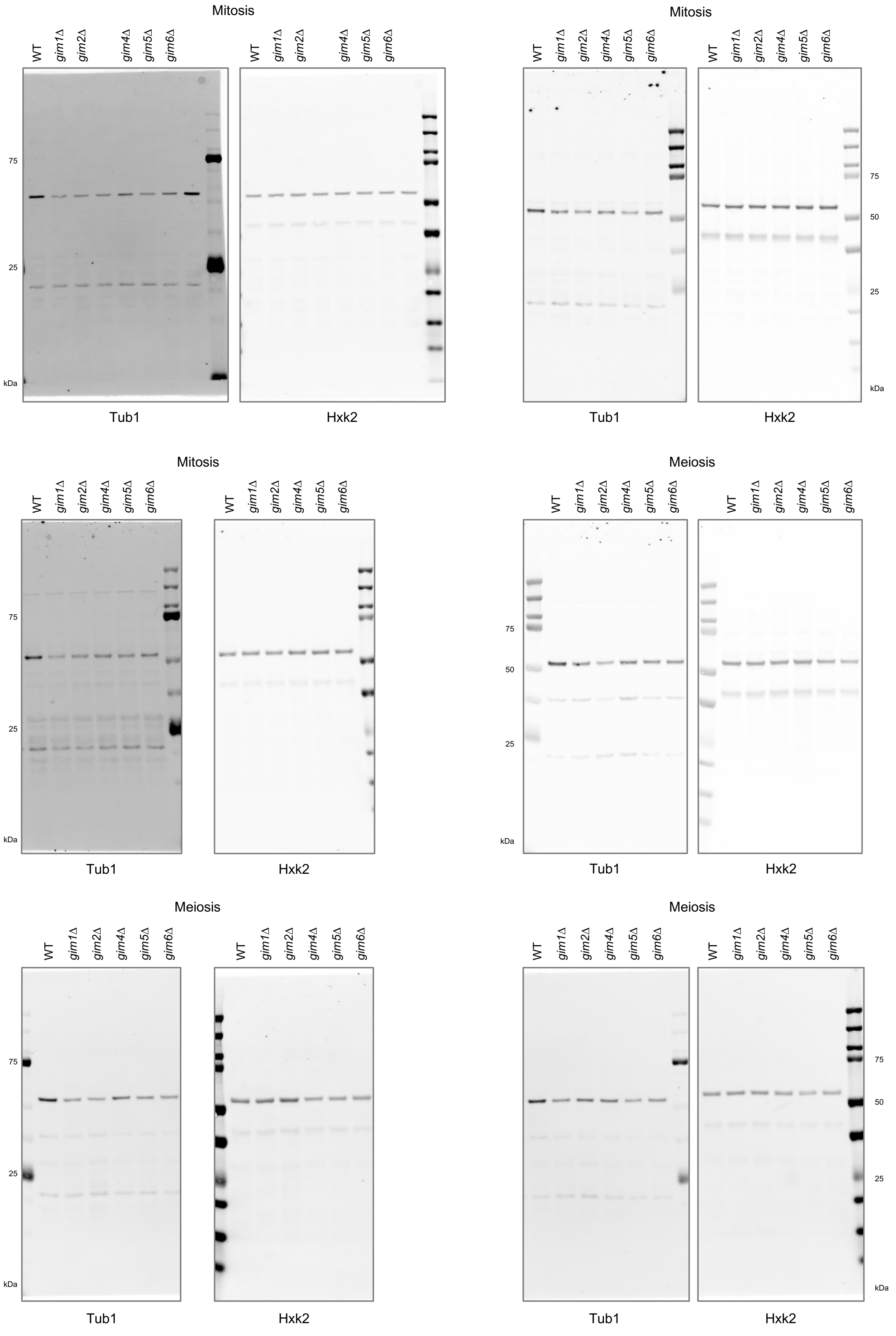
Mitosis
WT gim1Δ gim2Δ gim4Δ gim5Δ gim6Δ
75
25
kDa
Tub1
Hxk2
Mitosis
WT gim1Δ gim2Δ gim4Δ gim5Δ gim6Δ
75
50
25
kDa
Tub1
Hxk2
Mitosis
WT gim1Δ gim2Δ gim4Δ gim5Δ gim6Δ
75
25
kDa
Tub1
Hxk2
Meiosis
WT gim1Δ gim2Δ gim4Δ gim5Δ gim6Δ
75
50
25
kDa
Tub1
Hxk2
Meiosis
WT gim1Δ gim2Δ gim4Δ gim5Δ gim6Δ
75
25
kDa
Tub1
Hxk2
Meiosis
WT gim1Δ gim2Δ gim4Δ gim5Δ gim6Δ
75
50
25
kDa
Tub1
Hxk2

Figure S4D

Replicate 1

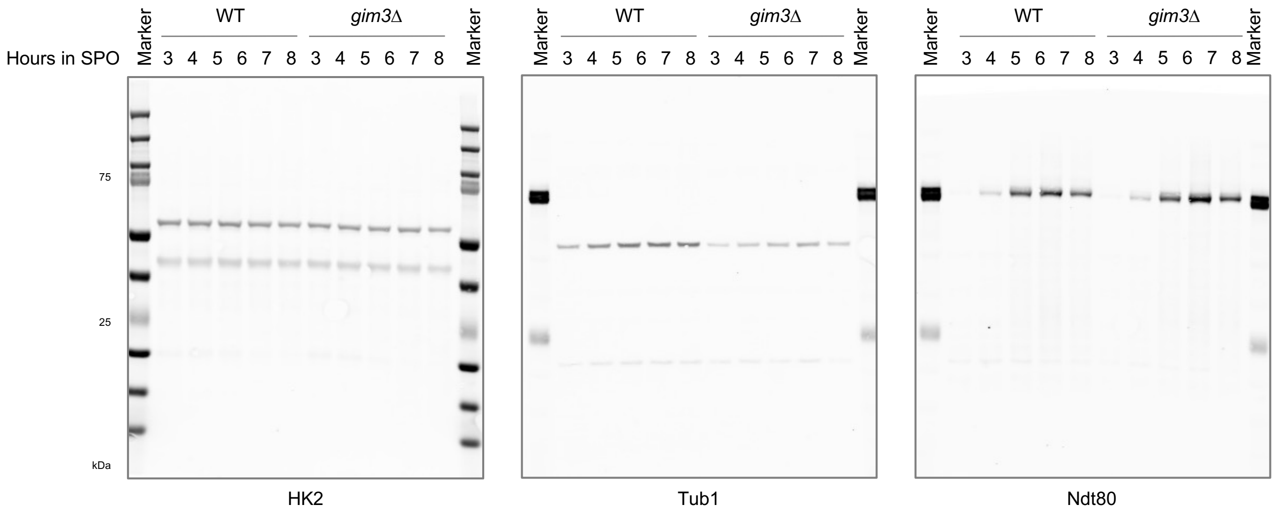

Replicate 2

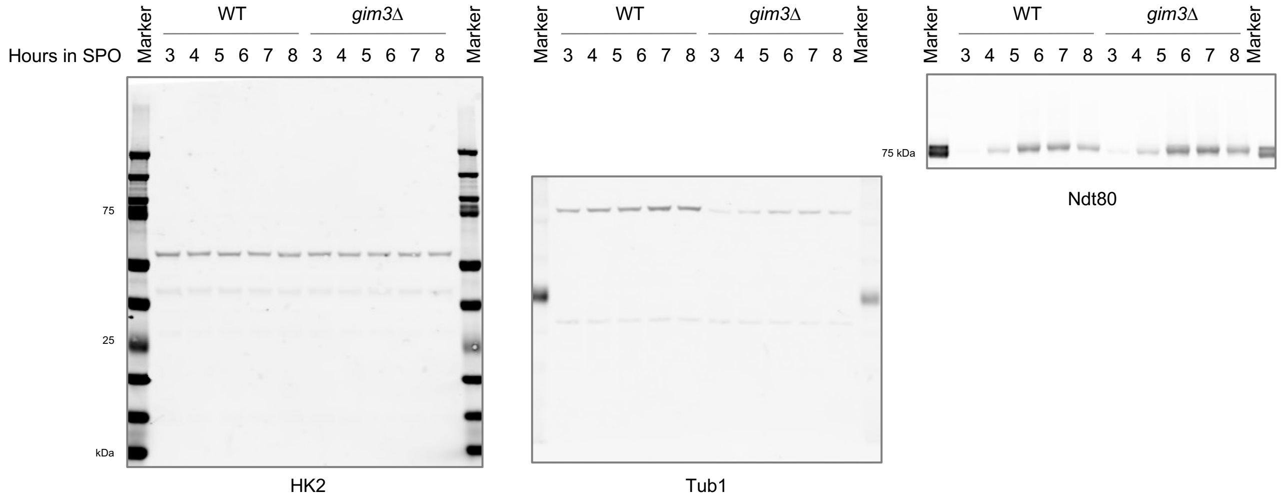

Replicate 3

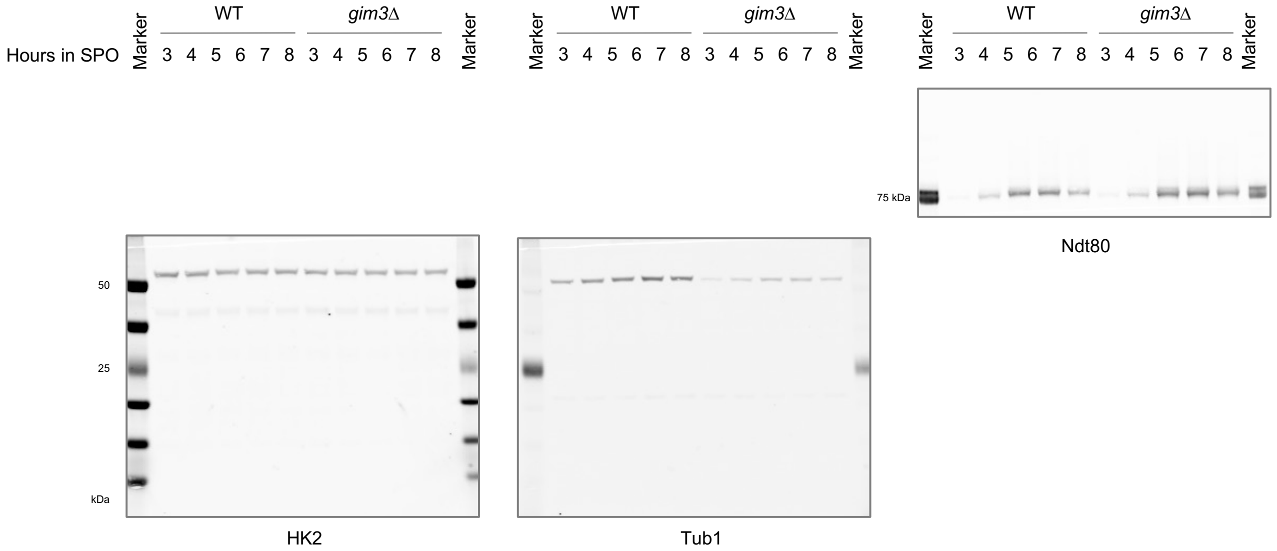

Figure S5B

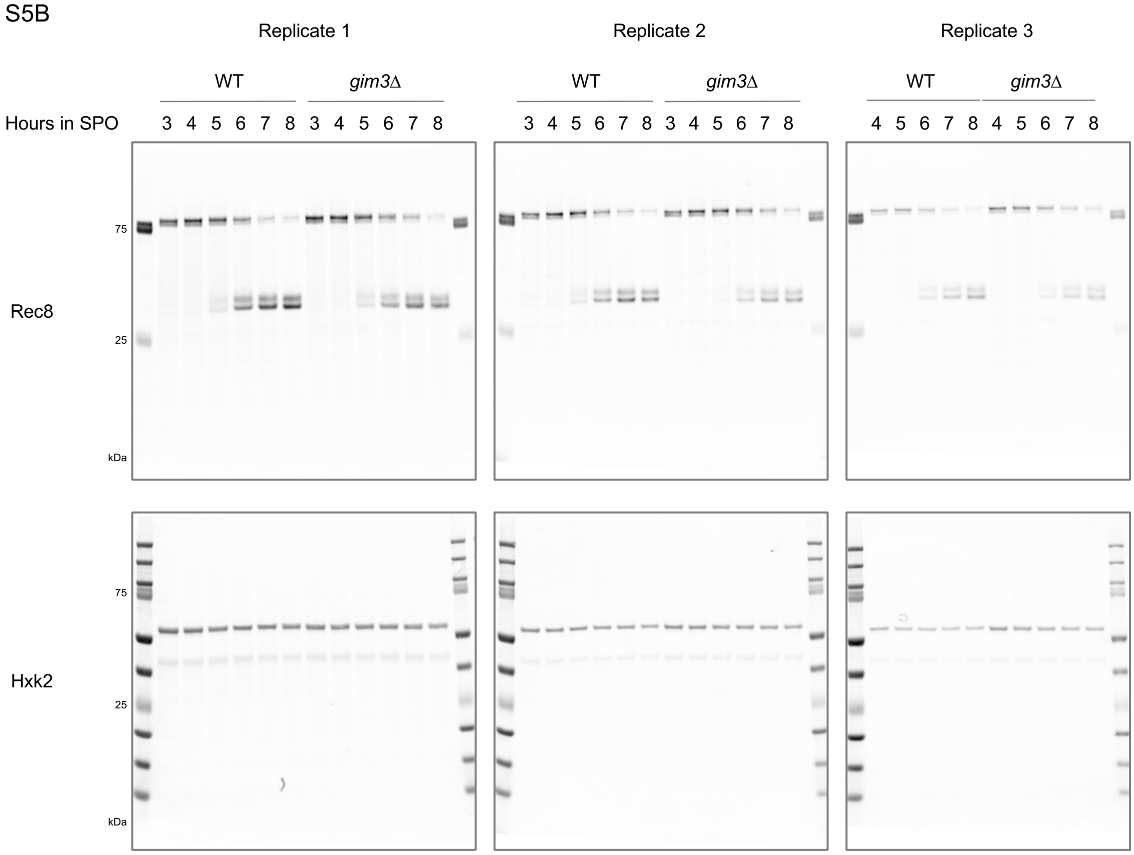

Figure S6G

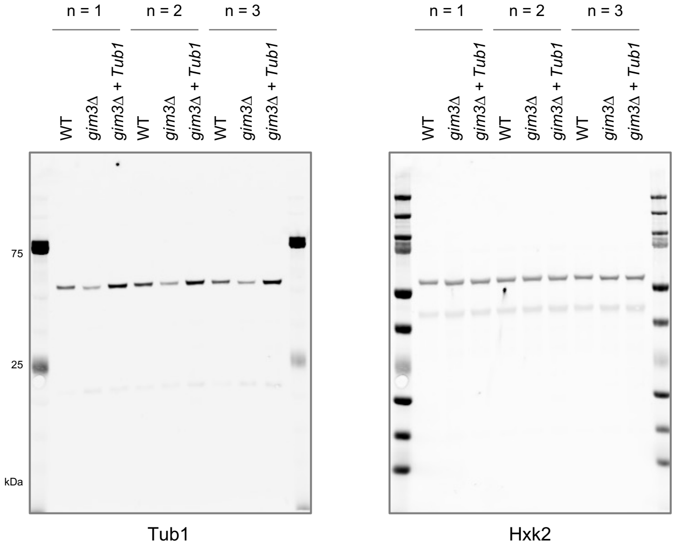

Supplement: Supplement 6 [file NIHPP2026.05.20.726416v1-supplement-6.pdf]
